# Docetaxel-loaded human serum albumin (HSA) nanoparticles: synthesis, characterization, and evaluation

**DOI:** 10.1186/s12938-019-0624-7

**Published:** 2019-01-31

**Authors:** Na Qu, Yating Sun, Yujing Li, Fei Hao, Pengyu Qiu, Lesheng Teng, Jing Xie, Yin Gao

**Affiliations:** 1 Key Laboratory for Molecular Enzymology and Engineering of Ministry of Education, School of Life Sciences, Jilin University, No.2699, Qianjin Street, Changchun, 130012 China; 2 State Key Laboratory of Long-acting and Targeted Drug Delivery System, Yantai, China

**Keywords:** Docetaxel, Human serum albumin, Nanoparticles, Self-assembly, Controlled release, Cytotoxicity, Maximum tolerated dose

## Abstract

**Background:**

Docetaxel (DTX) is an anticancer drug that is currently formulated with polysorbate 80 and ethanol (50:50, v/v) in clinical use. Unfortunately, this formulation causes hypersensitivity reactions, leading to severe side-effects, which have been primarily attributed to polysorbate 80.

**Methods:**

In this study, a DTX-loaded human serum albumin (HSA) nanoparticle (DTX-NP) was designed to overcome the hypersensitivity reactions that are induced by polysorbate 80. The methods of preparing the DTX-NPs have been optimized based on factors including the drug-to-HSA weight ratio, the duration of HSA incubation, and the choice of using a stabilizer. Synthesized DTX-NPs were characterized with regard to their particle diameters, drug loading capacities, and drug release kinetics. The morphology of the DTX-NPs was observed via scanning electron microscopy (SEM) and the successful preparation of DTX-NPs was confirmed via differential scanning calorimetry (DSC). The cytotoxicity and cellular uptake of DTX-NPs were investigated in the non-small cell lung cancer cell line A549 and the maximum tolerated dose (MTD) of DTX-NPs was evaluated via investigations with BALB/c mice.

**Results:**

The study showed that the loading capacity and the encapsulation efficiency of DTX-NPs prepared under the optimal conditions was 11.2 wt% and 63.1 wt%, respectively and the mean diameter was less than 200 nm, resulting in higher permeability and controlled release. Similar cytotoxicity against A549 cells was exhibited by the DTX-NPs in comparison to DTX alone while higher maximum tolerated dose (MTD) with the DTX-NPs (75 mg/kg) than with DTX (30 mg/kg) was demonstrated in mice, suggesting that the DTX-NPs prepared with HSA yielded similar anti-tumor activity but were accompanied by less systemic toxicity than solvent formulated DTX.

**Conclusions:**

DTX-NPs warrant further investigation and are promising candidates for clinical applications.
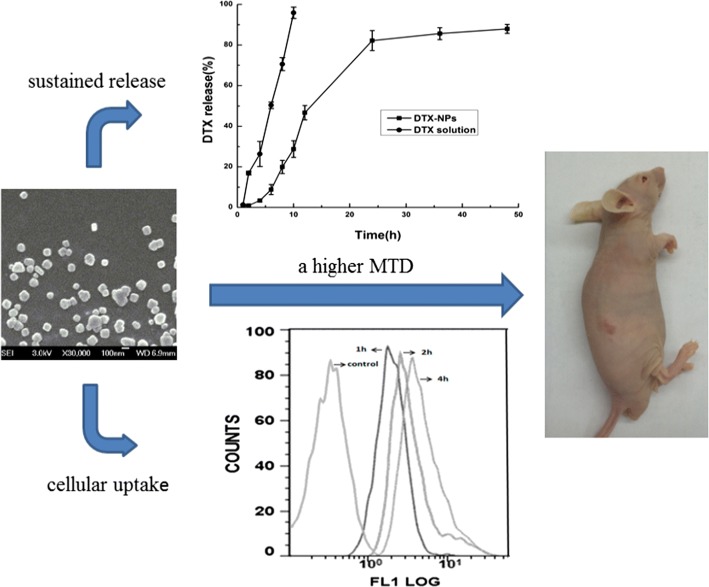

## Background

Lung cancer is the most common cause of cancer death among both men and women worldwide [1]. Docetaxel (DTX) is a Food and Drug Administration (FDA)-approved anti-cancer drug derived from taxoid, which leads to cell cycle arrest and death [2, 3]. This drug is currently used as a monotherapy or as a combination therapy to treat non-small cell lung cancer (NSCLC) [4] as well as other types of cancers including gastric [5], breast [6], ovarian [7] and prostate cancers [8]. Despite the clinically meaningful benefits of DTX, the anti-mitotic effect of DTX associated with high cytotoxicity and severe adverse effects has limited its therapeutic applications in terms of dosage and duration, especially among older patients [9]. Moreover, due to its hydrophobicity and low biocompatibility, the clinically administrated DTX is currently formulated with polysorbate 80 and ethanol (50:50, v/v), which causes hypersensitivity reactions in cancer patients, leading to a reduced uptake of DTX by tumor tissues and increasing its exposure to other body compartments, thus leading to harmful effects on normal tissues [10]. To address this problem, the development of alternative DTX formulations aiming to eliminate the side-effects and increase the therapeutic efficacy have become necessary and have thus attracted growing attention.

Recent studies have indicated that the incorporation of DTX into a drug delivery system effectively increases the half-life of DTX during systemic circulation in the comparison to the tween 80/ethanol-based clinical formulations, significantly enhances the anti-tumor effect, and reduces the systemic toxicity [11–13]. Liposomes and micelles with or without further modifications are widely accepted vehicles to increase the solubility of hydrophobic drugs, enhance the absorption, and extend the time of content release, thus altering the pharmacokinetic behavior and biodistribution of the drug cargos [11, 12, 14–17]. However, any foreign substance tends to induce immunogenic effects and the manufacture of liposomes has limitations in terms of high cost, quality assurance and the stability of liposomes during storage as well as difficulties in liposome sterilization, which in combination have impeded their clinical translations [18].

Nanoparticles are promising candidates for use as drug carriers [19]. They can be designed for drug delivery applications by controlling their surface properties, their particle diameters, and by regulating the expulsion of therapeutically active agents in order to achieve site specific drug action at therapeutically optimal rates [20]. Nanoparticle-based drug delivery platforms have various advantages, including the ability of offering targeted drug delivery to specific tissue sites in a controlled manner, protecting drugs from degradation and metabolism to enhance drug stability, offering sustained drug release over prolonged periods, and reducing undesirable side-effects [20]. Polymer-based nanoparticulate carriers have also provided promising candidates for drug delivery and have drawn increasing attention within the pharmaceutical field [10, 21–24]. Among them, the natural and synthetic polymers chitosan [10, 25, 26] and poly(lactide-*co*-glycolide) (PLGA), respectively [21, 24, 27] have often been conjugated with DTX due to their biocompatibility and biodegradability. However, either the rapid degradation of chitosan or slow degradation of PLGA have hindered their use as drug delivering nanoparticles (NPs) [28, 29].

As the most abundant plasma protein, human serum albumin (HSA) has emerged as a versatile drug delivery platform due to its good biocompatibility, non-toxic, and non-immunogenic properties as well as its biologically benign degradation file [30]. Numerous therapeutic drugs are delivered by HSA [31]. For example, the albumin paclitaxel (PTX) nanoparticle (Abraxane^®^) has already been approved by the FDA for the treatment of metastatic breast cancer [32, 33]. However, a major drawback of the conventional method for Abraxane^®^ preparation is the need for toxic chlorinated organic solvents [34]. In order to eliminate the risk of toxic organic solvent residues remaining in the nanoparticles after preparation, a greener salting-out method has been utilized to prepare DTX-HSA nanoparticles in this study and shows good prospects in terms of rendering the formulation safer for intravenous injection. In addition, the elimination of the use of organic solvents also can maintain the integrity and the biological activity of albumin, thus minimizing the occurrence of immunoreactivity issues in arising in the human body. Also, this method is more facile than the NabTM technique. Abraxane^®^ has been developed as a commercial PTX-HSA nanoparticle and is widely used in the treatment of cancer [35]. Despite this success, formulations incorporating a semisynthetic derivative of PTX, namely DTX, have not been explored. DTX has similar structural properties and exerts similar preclinical and clinical effects as those of PTX; but these drugs cannot be interchanged for the treatment of certain cancers [36]. By affecting cell growth, PTX primarily influences the G2/M phase, whereas DTX is more active during the S phase [37]. Therefore, the adoption of HSA nanoparticles as delivery vehicles for DTX merits further investigation.

In this study, a salting-out method for the preparation of DTX-loaded HSA-based NPs has been optimized. The DTX-HSA-NP formulation (DTX-NP) was characterized both in vitro and in vivo via various methods, with the aim of extending the use of DTX-NP in clinical practice.

## Results

### DTX-NPs preparation and characterization

As shown in Table 1, the diameters of the NPs increased in a directly proportional manner with respect to the drug-to-HSA ratio (w/w). However, only a slight difference in particle size was observed between the ratio of 0.1 and 0.2. The LC (wt%) at the drug-to-HSA ratio of 0.2 was 11.2 wt%, which was much higher than the value of 3.4 wt% observed at the drug-to-HSA ratio of 0.1. No significant difference in the LC was seen at drug-to-HSA ratios ranging from 0.2 to 0.4. Moreover, the highest EE (%) was obtained at the drug-to-HSA ratio of 0.2, which was 63.1 ± 1.9%, much higher than other preparations. Therefore, 0.2 was selected as the optimum drug-to-HSA ratio (w/w) for NP synthesis. Table 2 shows that the particle diameter increased with incubation time. Meanwhile, it was apparent that the duration of HSA incubation did not affect the LC and EE, and thus the best incubation time for protein chain assembly was 10 min. In addition, the effect of the stabilizer on the NP diameters was assessed. Particle sizes were measured before and after they had been subjected to freeze-drying treatment. Table 3 shows that the use of sodium gluconate yielded NPs with diameters smaller than 200 nm after freeze-drying treatment.

**Table 1:** Effect of the drug-to-HSA ratio (w/w)

Drug-to-HSA ratio (w/w)	Particle diameter (nm)	LC (%)	EE (%)
0.1	162.7 ± 1.7	3.4 ± 0.9	35.2 ± 0.9
0.2	163.2 ± 2.1	11.2 ± 1.4	63.1 ± 1.9
0.3	181.3 ± 1.9	12.1 ± 1.2	45.9 ± 1.6
0.4	198.8 ± 2.6	11.9 ± 1.9	33.8 ± 2.7

**Table 2:** Effect of the duration of HSA incubation

Duration of HSA incubation (min)	Particle size (nm)	LC (%)	EE (%)
10	142.8 ± 2.5	11.1 ± 1.3	62.4 ± 1.2
20	190.8 ± 2.1	10.7 ± 1.7	59.9 ± 2.0
30	193.3 ± 1.6	11.3 ± 1.4	63.7 ± 1.5

**Table 3:** Effect of the stabilizer

Stabilizer	Particle size (nm)	
	Before freeze-drying	After freeze-drying
Sodium tartrate	158.1 ± 3.1	1173.0 ± 10.2
Sodium gluconate	140.9 ± 2.9	139.4 ± 3.1

**Fig. 1:**
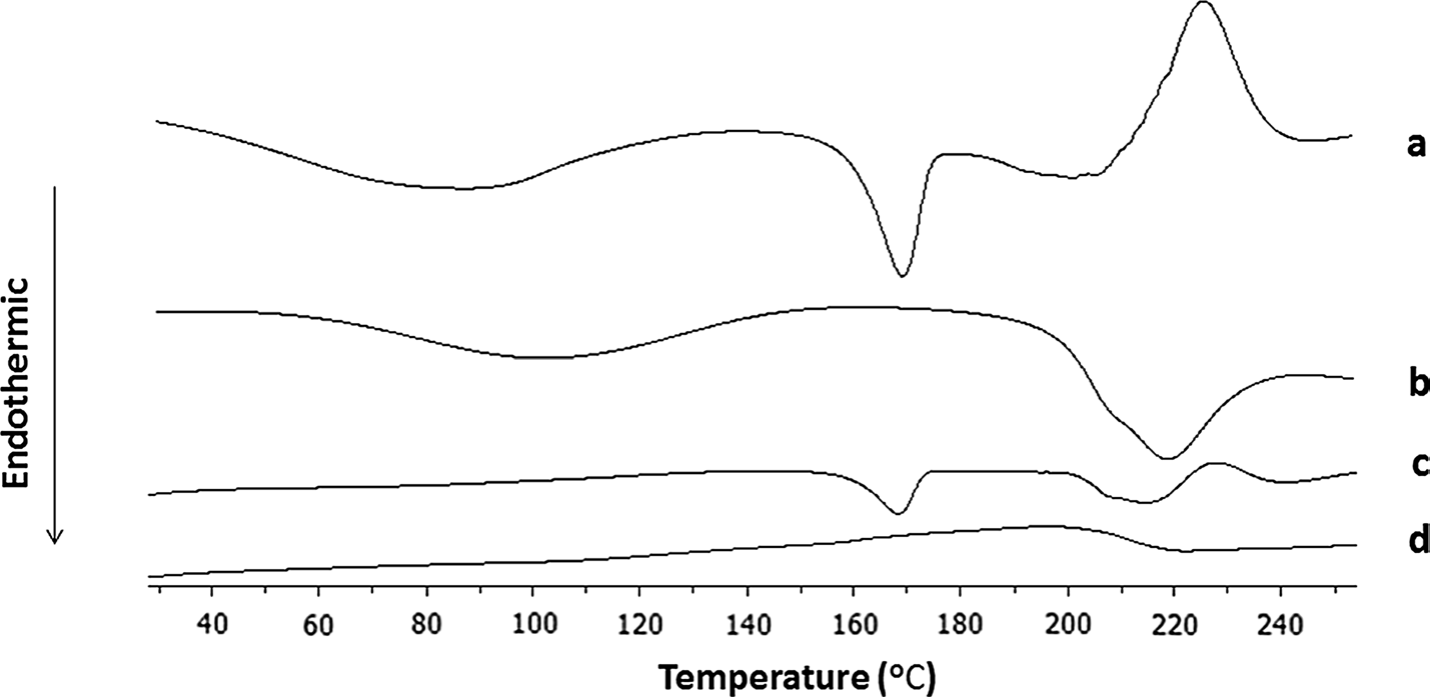
DSC thermograms of DTX (a), lyophilized HSA (b), physical mixtures of DTX and HSA (c), and lyophilized DTX-NPs (d)

**Fig. 2:**
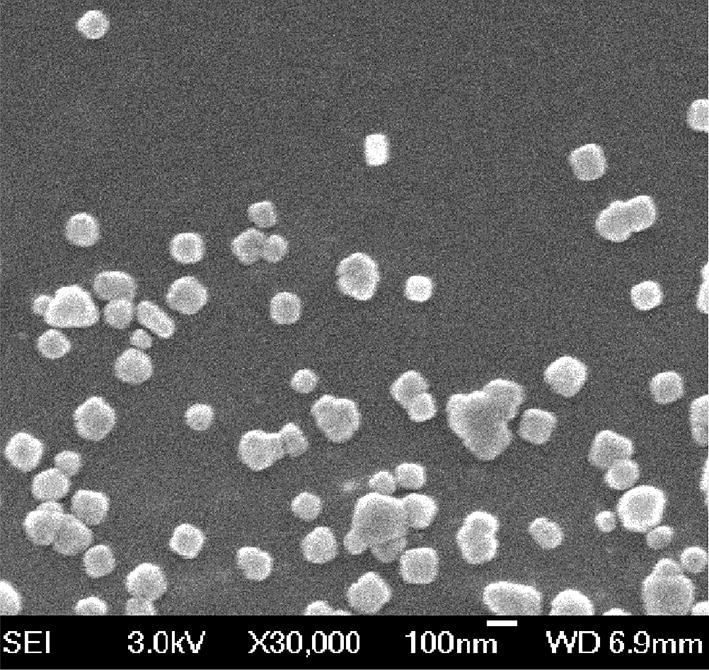
SEM image of DTX-NPs prepared under the optimized conditions

Figure 1 shows differential scanning calorimetry (DSC) spectra of the four samples. The melting temperature or endothermic peak of DTX was observed at 167 °C (Fig. 1a). The NPs did not show any characteristic crystalline peaks corresponding to DTX, thus confirming that the drug had become encapsulated within the DTX-NPs. Furthermore, an SEM image of the DTX-NPs is shown in Fig. 2. This image demonstrated that the DTX-NPs prepared under the optimal conditions had a spherical morphology, which is consistent with the results obtained from particle size analysis.

### In vitro release studies

**Fig. 3:**
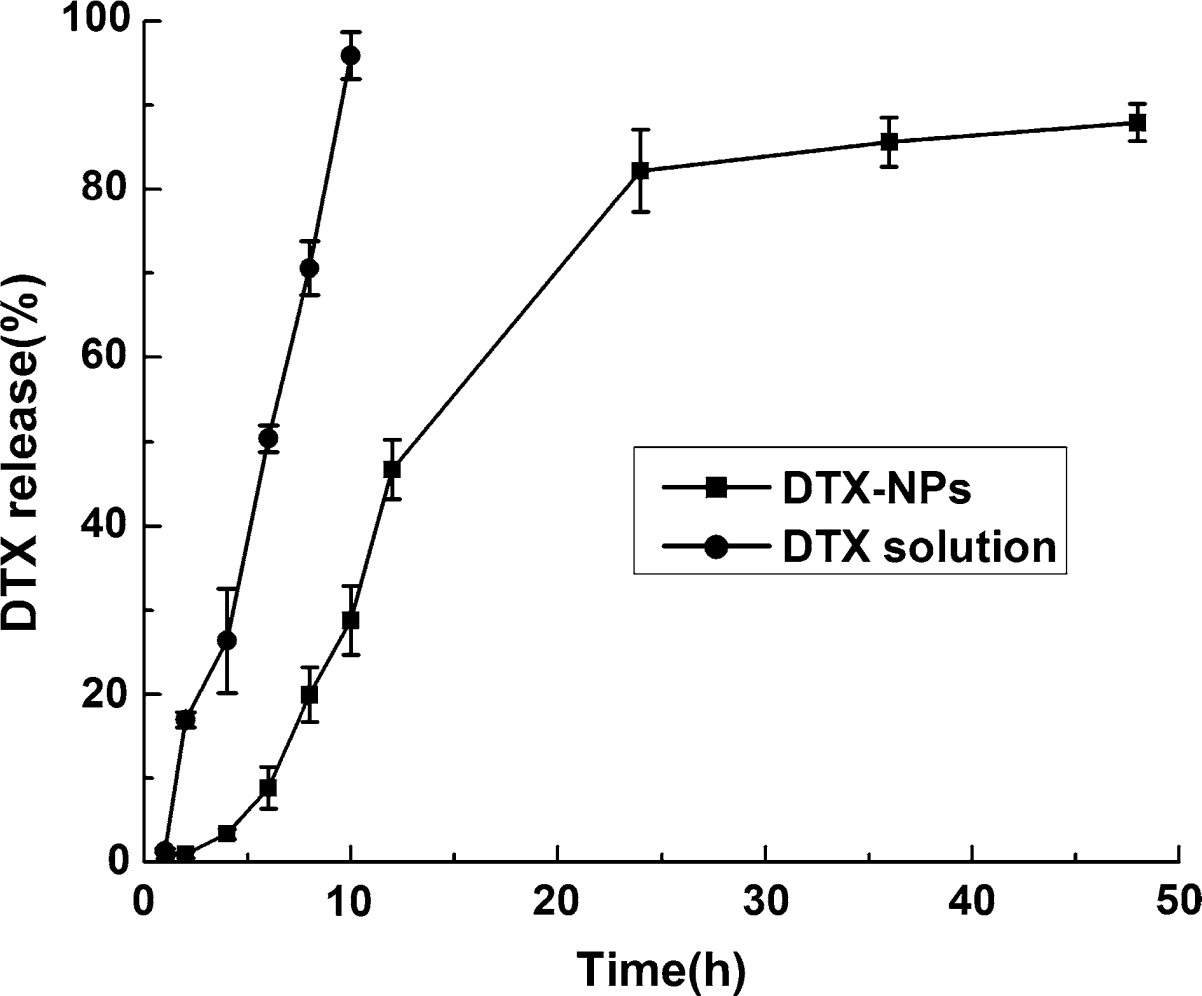
Release profile of DTX from DTX-solvent and DTX-NPs (*n *= 3)

The in vitro release kinetics exhibited by the DTX-NPs and free DTX were evaluated via the dialysis method as described in “In vitro release studies” section. Free DTX was rapidly released and reached a cumulative release of 95.92 ± 1.54% of the total drug within 10 h. In comparison with free DTX, a longer time was required for the release of DTX from in comparison with the DTX-NPs, (Fig. 3) demonstrated that the encapsulated drugs underwent a sustained release.

### Cellular uptake of DTX-NPs

**Fig. 4:**
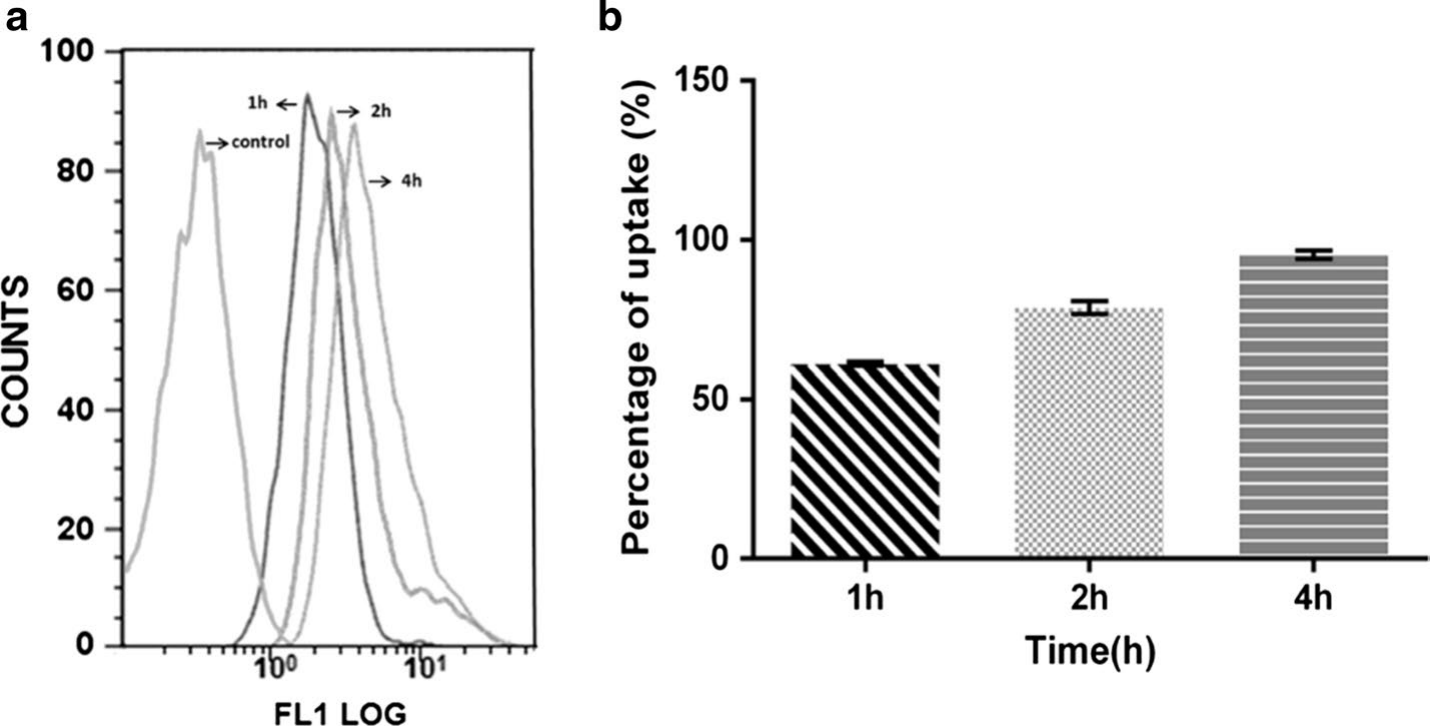
Quantification of the cellular uptake of FITC-DTX-NPs by flow cytometry at 1, 2 and 4 h, respectively. Flow cytometry histogram overlays (**a**). The percentage uptake of FITC-DTX-NPs at different incubation times (**b**)

**Fig. 5:**
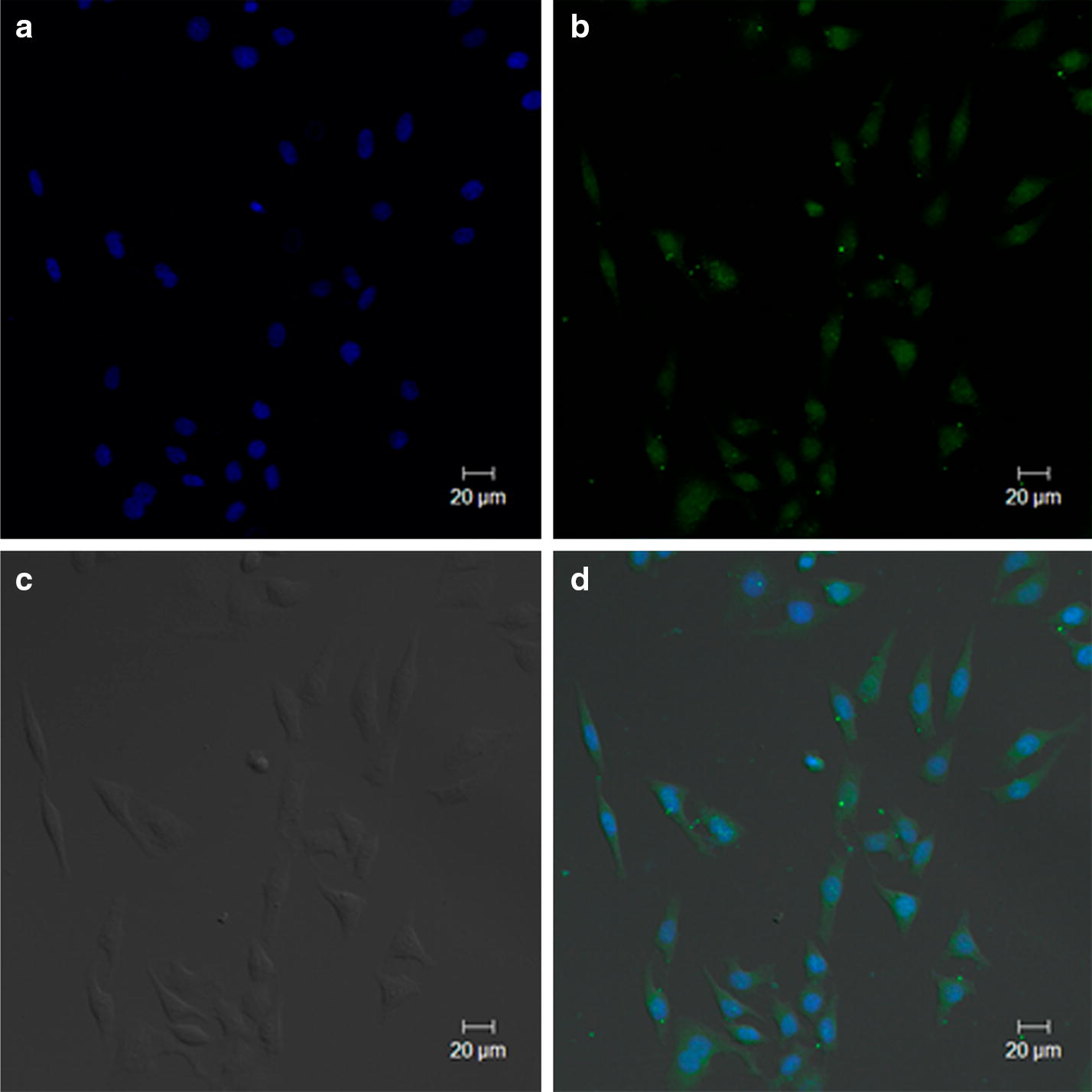
CLSM images of FITC-labeled DTX-NPs at 4 h in A549 cell line. The images correspond to: the blue channel showing nuclei that were stained with DAPI (**a**), the green channel showing DTX-NPs (**b**), a bright field image under phase contrast (**c**), and a merged image (**d**)

The cellular uptake of FITC-DTX-NPs by the A549 cells increased in parallel with an increase in the incubation time from 1, 2 and 4 h as determined via flow cytometry analysis (Fig. 4). The percentage of the cellular uptake reached the saturation state after 4 h of incubation time, as indicated in Fig. 4b. In addition, confocal laser scanning microscopy (CLSM) was employed to visualize the distribution of DTX-NPs within the A549 cells. As shown in the Fig. 5, after 4 h of incubation the fluorescence of FITC was clearly visible around the blue cell nucleus stained by DAPI, thus demonstrating that the FITC-DTX-NPs were internalized and dispersed throughout the cytoplasm [38].

### In vitro cytotoxicity

**Fig. 6:**
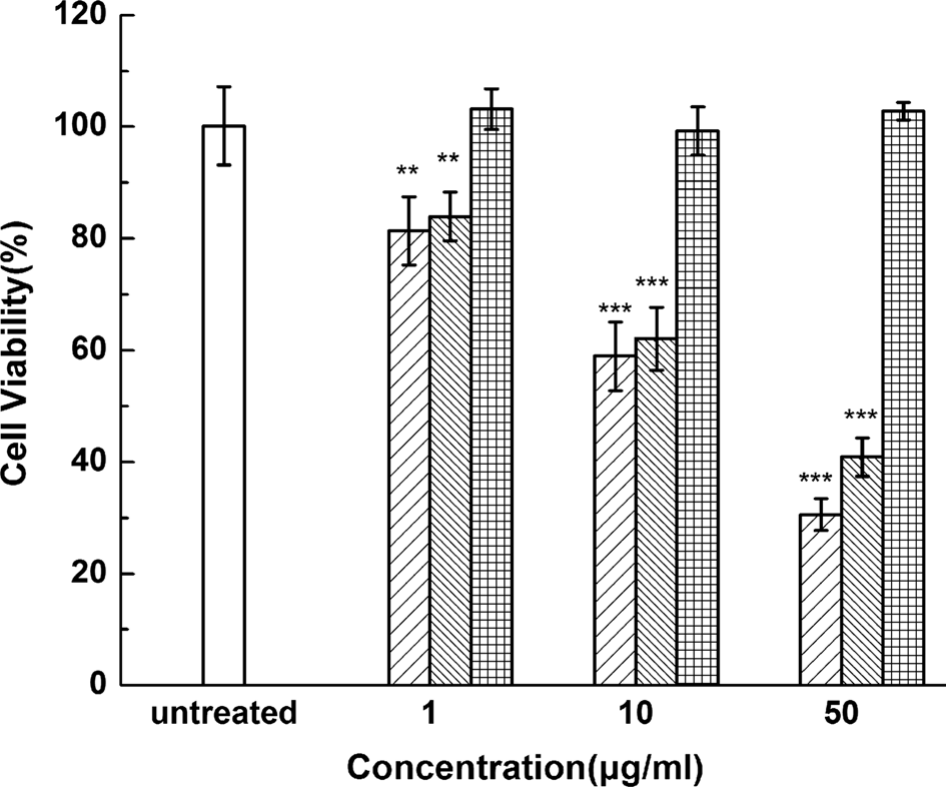
In vitro cytotoxicity study of a control (untreated) sample (  ), free DTX (  ), DTX-NPs (  ) and drug-free NPs (  ) against A549 cell line at various DTX concentrations. Data are expressed as mean ± SE (*n *= 6). **p < 0.01 and ***p < 0.001, in comparison with the control

The side-by-side experiments showed that the DTX-NPs and free DTX had similar cytotoxic effects when the concentrations of encapsulated and free DTX were 10 and 1 μg/mL, indicating that the DTX-NPs indeed had an anti-proliferative activity against A549 cells. As shown in Fig. 6, the drug-free HSA-NPs exhibited only a negligible cytotoxic effect, thus suggesting their potential suitability as drug carriers [39]. Meanwhile, the DTX-NPs and DTX exhibited nearly identical cytotoxic activity at equal concentrations, suggesting that the DTX-NPs had similar anti-tumor efficacy with lower systemic toxicity.

### Assessment of the maximum tolerated doses (MTDs)

**Fig. 7:**
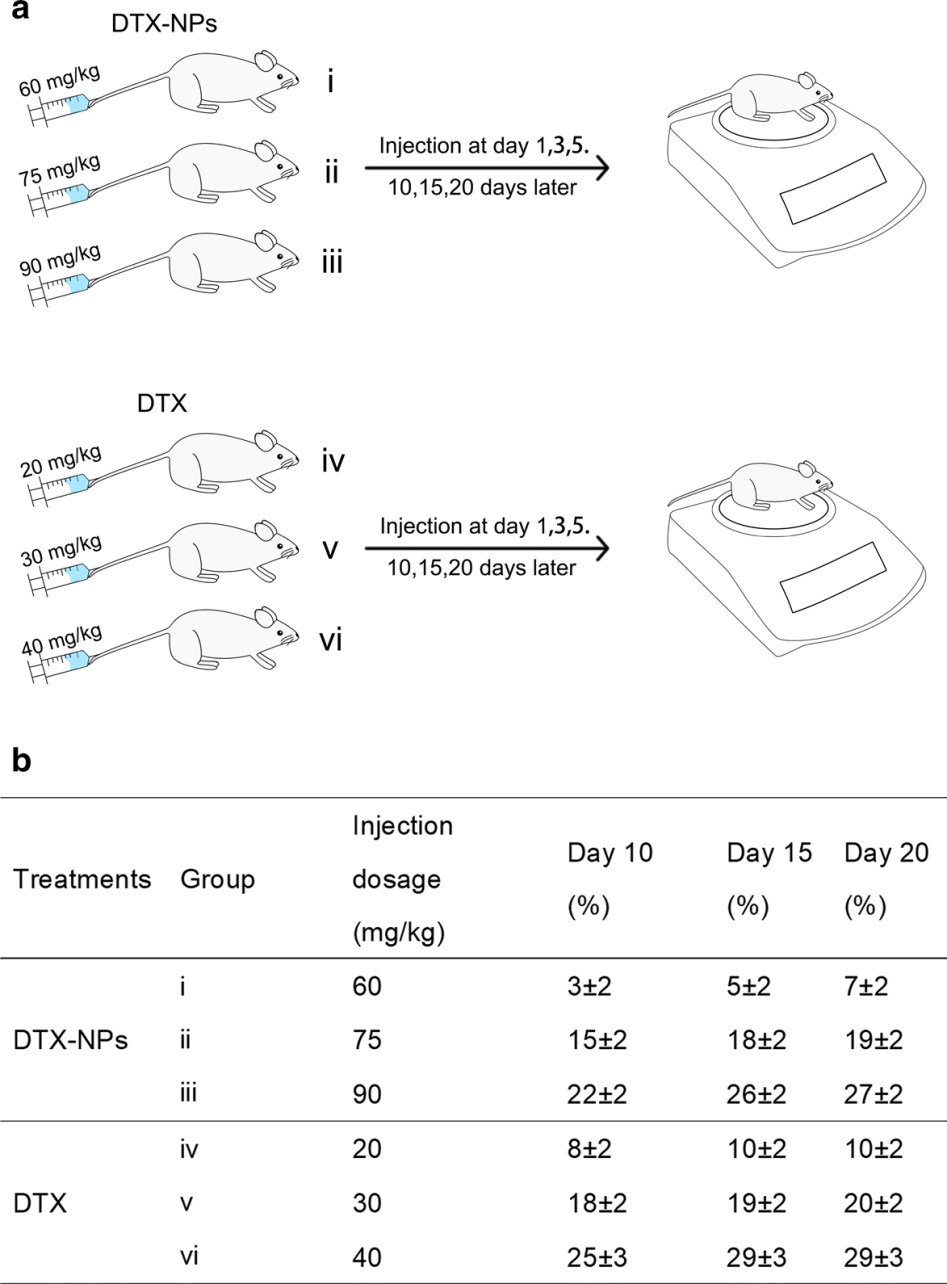
Depicted figure for assessment of the maximum tolerated dosage. Specific administration status and measurement procedure for DTX-NPs and DTX (**a**). The weight loss after administration of the mice (**b**)

Mice were treated with DTX-NPs and free DTX to establish the MTD (Fig. 7). According to the results, all of the mice that received injections experienced weight loss. The greatest average weight loss was observed on day 20 with the DTX-NP treatment, corresponding to 27 ± 2% for group iii (90 mg/kg of DTX-NPs) and 29 ± 3% for group vi (40 mg/kg). When the concentration of DTX-NPs was 75 mg/kg (group ii), the average weight loss was 19 ± 2%. This value was almost identical to that observed for group iv (20 ± 2%), which was treated with 30 mg/kg of free DTX. Meanwhile, with regards to groups i (60 mg/kg) and iv (20 mg/kg), the average weight losses were 7 ± 2% and 10 ± 2%, respectively. Therefore, the MTD of the DTX-NPs was almost 75 mg/kg while the MTD of free DTX was nearly 30 mg/kg. This demonstrated that mice can tolerate a higher concentration of encapsulated DTX than free DTX.

## Discussion

The use of Polysorbate 80 in formulations of Taxotere has been shown to cause hemolysis, hypersensitivity reactions, and fluid retention [11]. In this study, HSA NPs that did not require a cross-linking agent were developed as platforms for DTX delivery and the cellular uptake and cytotoxicity were investigated in the non-small cell lung cancer cell line A549. The method for DTX-NPs preparation was optimized with regard to the drug-to-HSA ratio (w/w), the incubation times of HSA, and the choice of stabilizer. The size of the DTX-NPs prepared via the optimized conditions remained below 200 nm, which was sufficiently small to facilitate prolonged NP circulation and penetration into the tumor tissue via the enhanced permeability and retention (EPR) effect. The LC (wt%) at the drug-to-HSA ratio of 0.2 was 11.2 ± 1.4%, which is higher than those obtained via other previous fabrication approaches, such as the reported albumin-based nanoparticles of curcumin (7.2 ± 2.5%) [40], paclitaxel (7.89 ± 1.31%) [41], tacrolimus (1.5 ± 0.1%) [42] and DTX (7%) [43]. Other nanoparticles of DTX, such as chitosan-based DTX nanoparticles [25], exhibit a similar LC (8–12%) with that achieved by this current approach and the solid lipid DTX nanoparticles show a much lower LC (1.90%, 1,92%) than the approach reported herein [44]. Meanwhile, the release kinetics exhibited by the DTX-NPs demonstrated that the encapsulated drugs underwent a sustained release, suggesting that they provide an effective means or prolonging the drug in circulation in vivo. In addition, the assessment of cellular uptake of FITC-DTX-NP by A549 cells demonstrated that the drug was successfully delivered into the cells. Although NPs are generally non-specifically internalized into cells via endocytosis or phagocytosis [45], HSA has been demonstrated to facilitate relatively high uptake in tumors and inflamed tissue [46]. Moreover, The drug-free HSA-NPs exhibited only a negligible cytotoxic effect on A549 cells, suggesting their potential suitability as drug carriers [39]. Therefore, the nearly identical cytotoxic activity exhibited by DTX-NPs and DTX at equal concentrations indicated that the use of HSA-NPs as a drug vehicle does not affect the anti-cancer effect of DTX, while maintaining similar anti-cancer activity in vitro but with lower cytotoxicity. MTD assessment further demonstrated that mice can tolerate a higher concentration of encapsulated DTX than free DTX. In general, the dosage of DTX used in the in vivo tumor inhibition experiment is less than 10 mg/kg, which can provide a significant tumor suppressing effect. For example, in the study of the anti-tumor effect of DTX-loaded polydopamine-modified TPGS-PLA nanoparticles for the treatment of liver cancer, a comparative investigation revealed that effective tumor suppression was achieved with the administration of 10 mg/kg of the free drug [47]. Moreover, a DTX-loaded BSA nanoparticle has been reported to overcome drug resistance and enhance the therapeutic efficacy with an administration dosage of 2 mg/kg [43]. In accordance with these results, the encapsulation of DTX by the albumin nanoparticles in this study greatly enhanced this drug’s biocompatibility. Last but not least, the HSA-NPs were non-toxic and safe for use as drug carriers while also exhibiting the prolonged circulation time and the aggregation-induced effect of enhanced permeability and retention (EPR effect). Therefore, DTX-NPs would exhibit better in vivo therapeutic effects than DTX alone, and they are promising candidates for clinical applications [48].

## Conclusion

A nanoparticle formulation of DTX with HSA was prepared via self-assembly under optimized conditions. These DTX-NPs had a favorable particle size below 200 nm and LC of ~ 11 wt%, which was desirable for intravenous injection. In vitro studies revealed that the DTX-NPs exhibited a sustained released of DTX which suggested that DTX-NPs are likely to prolong DTX in the circulation in vivo. Furthermore, a high cellular uptake was obtained with the DTX-NPs along with a higher MTD, suggesting that they offer promising anti-tumor activity with low systemic toxicity. In summary, these findings demonstrate that the use of HSA as a drug carrier for the preparation of DTX-NPs provides a promising platform for anti-cancer therapy (Fig. 7).

## Materials and methods

### Materials

Docetaxel (DTX) was purchased from Guilin Huiang Biopharmaceutical Co., Ltd. (Guilin, China). HSA (20%) was purchased from Octapharma (Vienna, Austria). Dimethylsulfoxide (DMSO), 3-[4,5-dimethylthiazol-2-yl]-2,5,-diphenyltetrazolium bromide (MTT), and 4′,6-diamidino-2-phenylindole (DAPI) were supplied by Shanghai Yuanye Bio-Technology Co., Ltd. (Shanghai, China). Fluorescein isothiocyanate (FITC) was obtained from Beijing Dingguo Bio-Technology Co., Ltd. (Beijing, China). Human lung cancer cell line A549 was acquired from American Type Culture Collection (ATCC, Logan, UT, USA). Organic solvents of chromatography grade were purchased from Sinopharm Chemical Reagent Beijing Co., Ltd. (Beijing, China).

### Methods

#### Preparation of the NPs

DTX-loaded HSA NPs (DTX-NPs) were prepared via a self-assembly method. Specifically, DTX (10–40 mg) was initially dissolved in 1 mL of ethyl alcohol. This solution was then mixed thoroughly with 0.65 mL of 12% disodium hydrogen phosphate and 100 mg of HSA, which had been pre-heated to 65 °C. After incubation for 3 min at 65 °C, the solution was slowly injected into 50 mL of 0.05 M sodium tartrate or sodium gluconate buffer with stirring (900 rpm) at 65 °C. This solution was immediately chilled in an ice bath. The remaining unencapsulated DTX was subsequently removed via ultrafiltration. The samples were freeze-dried without the use of additional cryoprotectants.

#### Single-factor optimization of DTX-NP preparation

Three parameters, including the drug-to-HSA (w/w) ratio, the duration of HSA incubation, and the choice of stabilizer, were selected as independent variables. The selected drug-to-HSA (w/w) ratios were 10, 20, 30, and 40 mg/mL. Meanwhile, the incubation times of HSA were 10, 20, and 30 min. In addition, the choice of stabilizer included either sodium tartrate or sodium gluconate buffer. The preparation of DTX-NPs is described above in “Preparation of the NPs” section. The effects of the three factors on the particle diameter and loading capacity (LC) of DTX-NPs were independently evaluated. Finally, DTX-NPs that were synthesized via the optimal conditions were used in the subsequent investigations.

#### Particle diameter and morphological characterization

The mean particle diameters of the NPs were determined via dynamic light scattering (DLS) measurements using a Zetasizer Nano ZS 90 (Malvern Instruments, Ltd., Malvern, UK) [49]. The morphologies of the NPs were investigated via scanning electron microscopy (SEM) using a JSM-6700F microscope manufactured by JEOL (Tokyo, Japan). One droplet of diluted suspension was deposited onto a patch of silicon wafer and dried at room temperature. The sample was subsequently sputter-coated with platinum and then examined by SEM.

#### Determination of the drug LC and EE

The drug concentration was initially determined by high-performance liquid chromatography (HPLC) using a Shimadzu-20AD system (Kyoto, Japan). The HPLC system was equipped with a Diamonsil C18 reverse-phase column (particle size 5 µm, 4.6 × 150 mm) manufactured by Dikma Technologies (Beijing, China). The detection wavelength was set at 232 nm and the column temperature was maintained at 40 °C. A mixture of 0.043 M ammonium acetate and acetonitrile (45:55, v/v) was used as the mobile phase at a flow rate of 1 mL/min. The drug content in the DTX-NPs was then determined via a literature, which is briefly summarized as follows [50]. The NPs were diluted in acetonitrile and sonicated for 15 min. The supernatant obtained after centrifugation at 12,000 rpm for 10 min was then injected into the HPLC system in a volume of 20 μL to quantify the amount of DTX. The equations used for calculating LC (%) and EE (%) are as following.

$$ {\text{LC}}\,\left( \% \right) = \frac{{{\text{The}}\;{\text{amount}}\;{\text{of}}\;{\text{DTX}}}}{{{\text{The}}\;{\text{weight}}\;{\text{of}}\;{\text{lyophilized}}\;{\text{nanoparticles}}}} \times 100\% $$

$$ {\text{EE}}\,\left( \% \right) = \frac{{{\text{DTX}}\;{\text{total}} - {\text{DTX}}\;{\text{free}}}}{{{\text{DTX}}\;{\text{total}}}} \times 100\% $$

#### In vitro release studies [51]

To investigate the kinetics of DTX release from the DTX-NPs, a suspension of DTX-NPs containing 2 mg of DTX was transferred into a dialysis bag with a MWCM of 8000 Da. This dialysis bag was subsequently placed into 80 mL of release medium (PBS) containing 0.5% v/v Tween 80. The temperature was maintained at 37.0 ± 0.5 °C and the medium was stirred at a speed of 100 rpm. DTX (1 mg/mL) which had been dissolved in polysorbate 80 and 13% (w/w) ethanol (free DTX) was used as a control and was treated in the same manner). During a period of 48 h, the surrounding environment was maintained by replacing 0.5 mL of the release medium with an equal volume of fresh medium at regular intervals. The extraction solution was solubilized in 500 μL of acetonitrile and assayed by HPLC.

#### Differential scanning calorimetry (DSC)

Thermograms were obtained via DSC measurements. The samples of DTX, lyophilized HSA, physical mixtures of DTX and HSA, and lyophilized DTX-NPs were placed in sealed aluminum pans. During these measurements an empty pan was also used as a reference. These samples were heated from 20 to 250 °C at a rate of 10 °C/min under a flow of nitrogen gas (20 mL/min). Each sample was prepared and analyzed in triplicate.

#### In vitro cytotoxicity assays

The cytotoxicity of the DTX-NPs was determined in A549 cells using the MTT assays. Briefly, the cells were plated at a density of 12,000 cells per well and incubated with 100 µL of serial dilutions of DTX-NPs (DTX concentrations of 1, 10 and 50 μg/mL), free DTX (dissolved in polysorbate 80 and 13% (w/w) ethanol) and drug-free NPs. After another 24 h, 20 µL of MTT (5 mg/ml in PBS, pH = 7.4) was added and incubated at 37 °C for 4 h. The medium was then removed and 100 µL of DMSO was added into each well to dissolve the crystals. OD was then measured at 490 nm using a microplate reader (Synergy4, multi-mode microplate reader, BioTek, Winooski, VT, USA).

#### Cellular uptake determination of DTX-NPs in vitro

To detect the uptake of the NPs by A549 cells, FITC-labeled HSA was used to prepare the NPs (FITC-DTX-NPs). Cells were seeded at a density of 7 × 10^4^ cells per well onto 24-well plates and incubated with FITC-DTX-NPs for 1, 2, or 4 h. Untreated cells were used as a control. After the cells had been gently washed twice with PBS, they were re-suspended and fixed with 4% paraformaldehyde and analyzed using a EPICS XL flow cytometer (Beckman Coulter Corp, Tokyo, Japan) to measure the fluorescence intensities.

To prepare the samples for fluorescence microscopy imaging, 7 × 10^4^ cells per well were incubated with FITC-DTX-NPs for 4 h and fixed with 4% paraformaldehyde. The nuclei were stained with DAPI for 3 min. Fluorescence microscopy images were obtained using a Zeiss 710 LSMNLO Confocal Microscope (Carl Zeiss; Jena, Germany).

#### Assessment of the maximum tolerated dosage (MTD)

Male BALB/c Mice were purchased from Beijing Vital River Laboratory Animal Technology Co. Ltd. (Beijing, China). Prior to the experiments, the entire animal protocol was reviewed and approved by the Institution Animal Ethics Committee (Jilin University, license No.SCXK-(JI) 2011–0003) and we adhered to the Guidelines on Humane Treatment to Lab Animals (published in 2009) [39]. The mice were randomly separated into six groups (*n* = 6). DTX-NPs were dispersed in 0.9% saline solution with the DTX concentrations to be 60, 75 and 90 mg/kg (corresponding to groups i, ii and iii, respectively). Meanwhile, free DTX was dispersed into Tween 80 and 13% (w/w) ethanol in water at a ratio of 1:3 (w/w), at DTX concentrations of 20, 30 and 40 mg/kg to yield groups iv, v and vi, respectively. The mice were injected intravenously via their tail vein on days 1, 3 and 5. They were weighed prior to each injection and they continued to be weighed on days 10, 15 and 20. The dosage that resulted in a 20% loss in weight was defined as the MTD [52].

## Abbreviations

HSA: human serum albumin
DTX-NPs: docetaxel-loaded HSA nanoparticles
DTX: docetaxel
DSC: differential scanning calorimetry
EE: encapsulation efficiency
MTD: maximum tolerated dose
NPs: nanoparticles
DMSO: dimethylsulfoxide
MTT: 3-[4,5-dimethylthiazol-2-yl]-2,5,-diphenyltetrazolium bromide
DAPI: 4′,6-diamidino-2-phenylindole
FITC: fluorescein isothiocyanate
LC: loading capacity
SEM: scanning electron microscopy
HPLC: high-performance liquid chromatography
PBS: phosphate buffered saline
